# Midwifery hurdles: Navigating tuberculosis screening challenges in South Africa

**DOI:** 10.4102/curationis.v47i1.2533

**Published:** 2024-06-10

**Authors:** Violet M. Chewe, Sisinyana H. Khunou

**Affiliations:** 1 Department of Advanced Nursing Science, Faculty of Health Sciences, University of Venda, Thohoyandou, South Africa; 2 Department of Health Studies, College of Human Sciences, University of South Africa, Pretoria, South Africa

**Keywords:** midwives, challenges, pregnant women, screening, tuberculosis

## Abstract

**Background:**

In South Africa, screening for tuberculosis during pregnancy is a serious challenge. Tuberculosis is one of the leading indirect causes of mortality in pregnant women.

**Objectives:**

The objective of the study was to explore the challenges experienced by midwives regarding tuberculosis in pregnant women.

**Method:**

A qualitative exploratory research method was used to conduct the study. The study population comprised midwives who worked at primary healthcare clinics in the selected local area, Capricorn District, Limpopo province. Purposive non-probability sampling was used to select 10 participants. Data from participants were acquired using in-depth individual semi-structured interviews. Data analysis was carried out using manual thematic analysis following Tesch’s technique.

**Results:**

The outcomes of this study included midwives knowing their roles regarding tuberculosis screening among pregnant women. They further highlighted their challenges while screening tuberculosis in pregnant women, such as shortage of screening tools, withholding of tuberculosis information, and language barrier.

**Conclusion:**

Midwives should have the necessary equipment and be trained in various languages used in the province to improve tuberculosis screening among all pregnant women.

**Contribution:**

Infected pregnant women and their unborn children’s health can be improved by tuberculosis screening.

## Introduction

Individuals suffering from tuberculosis (TB) usually present with fever, weight loss, and a chronic productive cough (National Department of Health [NDoH] 2016:119). However, in acute infections, the symptoms normally resolve in 3 weeks or less. Therefore, if an individual’s cough persists after taking antibiotics for over 3 weeks, they should be screened for TB.

The World Health Organization (WHO) (2015:2) estimates that 1.2 million new TB cases were detected worldwide in 2015. According to WHO (2023:2), 10m people worldwide were diagnosed with TB in 2018, of which 3.2m were female and mostly of reproductive age. World Health Organisation further states that pregnant women and their unborn babies are at greater risk from untreated TB than from treatment. Untreated, TB infection during pregnancy may result in low birthweight babies and, in rare cases, congenital TB (Centre for Disease Control and Prevention 2023:3). Therefore, WHO (2023:3), recommends that pregnant women be screened and treatment be initiated as soon as there is a moderate to high possibility of TB infection.

In sub-Saharan African (SSA) countries, TB persists as an existential threat despite efforts to curb the epidemic (Adeizera, Abba & Okpapi 2014:11). Even though Palombi and Moramarco (2018:12) indicate that TB infection during pregnancy is a severe health risk that kills millions of people annually in SSA nations, the study by Glaw et al. (2019:7) reported a decline in TB among pregnant women in SSA.

In SA, non-pregnancy-related infections (NPRIs) continue to be the leading cause of maternal mortality, accounting for more than half of cases; TB is the most prevalent infection (NDoH 2023:4). According to Odayar et al. (2018:764), the prevalence of TB infection during pregnancy in SA remains elevated despite available screenings and treatment options.

Despite claims that primary healthcare (PHC) facilities screen pregnant women for TB, Limpopo is one of the SA provinces where maternal mortality from TB infection during pregnancy is a major cause of death (NDoH 2016:47). According to the Saving Mothers’ report, NDoH (2023:12), Limpopo province had 157 maternal deaths in 2017–2019, of which 48.6% were because of NPRI with HIV complicated by TB being the leading cause. There could be catastrophic effects if TB is not diagnosed, as there is a higher risk of miscarriage, premature birth, and maternal death (Pop et al. 2021:168). The purpose of the study was to explore the challenges faced by midwives during TB screening of pregnant women in Limpopo province.

## Research methods and design

### Study design

A qualitative explorative-descriptive design was employed for this study. This method is a well-structured approach used to comprehend human health, practices, health behaviour, and services (Brink, Van der Walt & Van Rensburg 2012:96), which assisted the researcher in investigating the challenges midwives confront when screening pregnant women for TB. The study’s design was appropriate as it addressed the midwives’ perspectives and limitations about TB screening for pregnant women.

### Setting

The study was conducted in selected clinics under Kganya Local Area within the Polokwane municipality in Capricorn district, Limpopo. This area had seven PHC clinics 10 km–30 km apart, offering maternity health services. Each clinic saw about 67 pregnant women monthly. Maternal mortality in Limpopo is 106/100 000 live births, which is above the WHO recommendation of 70/1000, with Capricorn being the highest with 67/100 (NDoH 2023:47)

### Population

All the midwives and accoucheurs presently employed at the selected local area clinics in the Capricorn District of the Limpopo province made up the population for this study. There are seven clinics in the selected local area. Forty-two midwives worked in the selected local region during the research, making up the entire population. A minimum of 5 years of experience working with pregnant women was required for midwives to be eligible to participate in the study. The midwives who did not consent to participate in the research were excluded. The distribution of the population is shown in Table 1.

**TABLE 1:** Study population.

Name of clinic	Number of midwives	Number of accoucheurs
Clinic A	5	1
Clinic B	6	0
Clinic C	9	1
Clinic D	5	0
Clinic E	6	1
Clinic F	6	1
Clinic G	5	0
**Total**	**42**	**4**

*Source:* Chewe, V.M., 2021, ‘Tuberculosis screening among human immune deficiency virus positive pregnant women in Limpopo Province’, MA Dissertation, University of South Africa, Pretoria

### Sample size

For this study, non-probability purposive sampling was employed. The researcher used purposeful sampling to get information on TB screening from midwives working at clinics in the selected local area. Participants were selected based on their preparedness, willingness, and participation interest. Data saturation was used to establish the sample size. The research reached data saturation when more interviews yielded no new information. A total number of 10 participants were interviewed for the study.

### Data collection

This study’s data-gathering method was a virtual one-on-one interview. Restrictions related to coronavirus disease during data collection led to virtual interviews. The interview made it easier for the researcher to get comprehensive information from the participants. The participants could identify their challenges when screening TB in pregnant women. In addition, to enable the collection of rich data, the researcher was permitted to probe and alter questions throughout the interview.

### Data analysis

Data analysis was conducted using Tesch’s eight processes to uncover and create themes and subthemes (Green & Thorogood 2018:251). Data organisation helped the researcher categorise, focus, remove, and arrange data to reach conclusions and verification. The transcripts, field notes, and audiotapes were stored securely to lessen the scientific pressure of data analysis and minimise contamination. The acquired data were listened to, word-for-word transcribed, and perceptions were recorded as they occurred to ensure reliability. As a result, the researcher carefully and repeatedly read the participant’s transcripts until she comprehended them. The researcher safeguarded and kept all data records in its original format.

### Ethical considerations

The University of South Africa Research Committee Provided ethical clearance (HSHDC/934/2019), and the Limpopo Department of Health (LP-202001-015) permitted the study. Managers from the clinic and the Capricorn District also gave their approval. The participants were given information about the study and a brochure before the study. All participants who volunteered to participate in the study gave their informed consent. During the data-collection process, all moral guidelines were observed. Principles of ethics such as autonomy, which gave participants the free will to decide whether to participate in the study, were followed. Pseudonyms P1 through P10 were assigned to participants to preserve their anonymity. The study also adhered to the principles of beneficence and maleficence by safeguarding participants from harm or suffering during the data-collection process. The participants were further informed that should they decide to terminate the study, they would not be held responsible. Finally, the justice principle was upheld. Participants were identified as P1 through P10 to maintain their anonymity and confidentiality.

## Results

Data saturation was attained after interviewing nine midwives and one accoucheur. The age of the participants ranged between 30 years and 48 years. They have between 6 years and 17 years of combined experience working with pregnant women. One male and nine female individuals were involved in the study. Participant number (P and number), gender (female [F] or male [M]) and clinic name (clinic [C] and alphabetical letter according to Table 1) were assigned as participants’ identification. Table 2 provides information about the participants’ sociodemographic.

**TABLE 2:** Socio-demographic information of the participants.

Participant no.	Age (years)	Gender	Academic qualification	Experience (years)
P1	37	Female	Midwife	10
P2	35	Female	Midwife	6
P3	43	Male	Accoucheur	12
P4	45	Female	Midwife	9
P5	35	Female	Midwife	10
P6	33	Female	Midwife	7
P7	34	Female	Midwife	7
P8	48	Female	Midwife	17
P9	40	Female	Midwife	11
P10	30	Female	Midwife	6

*Source:* Chewe, V.M., 2021, ‘Tuberculosis screening among human immune deficiency virus positive pregnant women in Limpopo Province’, MA Dissertation, University of South Africa, Pretoria

Roles and responsibilities and TB screening were the two primary themes that emerged as midwives’ and accoucheur’s experiences of TB screening among pregnant women. Forty-five emergent codes led to the themes and sub-themes. Subsequent sections detail each theme. In Table 3, the themes and sub-themes are described.

**TABLE 3:** Themes and sub-themes.

Theme	Sub-themes
1. Roles and responsibilities	1.1 Adequate TB screening 1.2 Embracing proper implementation of TB guidelines 1.3 Counselling and health education
2. TB screening challenges	2.1 Shortage of working tools 2.2 Withholding of TB symptoms information 2.3 Language barrier to international pregnant women

*Source:* Chewe, V.M., 2021, ‘Tuberculosis screening among human immune deficiency virus positive pregnant women in Limpopo Province’, MA Dissertation, University of South Africa, Pretoria

TB, tuberculosis.

### Roles and responsibilities

The participants demonstrated that they knew their obligations and responsibilities concerning TB screening of pregnant women. Three subthemes emerged: adequate TB screening, embracing proper implementation of TB guidelines, and counselling and health education.

#### Adequate tuberculosis screening

Participants expressed support for TB pregnancy screening. According to the participants, all pregnant women who visit the facility for antenatal care are screened for TB. If found to be symptomatic, their sputum is collected for gene-expert testing. This participant’s expressions are as follows:

‘In general, my view is that any pregnant woman who comes to this facility, we try to screen them and then ask them questions about TB or, actually what can I say, about symptoms if ever they are having symptoms and then we do test every pregnant woman.’ (P2, M, CE)

‘According to me, every pregnant woman needs to be screened each time she visits the clinic.’ (P5, F, CD)

#### Embracing proper implementation of tuberculosis guidelines

Guidelines and protocols are crucial in guiding midwives in screening a pregnant woman for TB. The participants shared their understanding of the various TB guidelines that are available and how important they are. It was observed that the participants apply TB guidelines in their facilities. The next quotations demonstrate this:

‘We follow the guidelines and policies, they direct us, they direct us if we ever face difficulties managing a pregnant woman because we don’t know how to.’ (P10, F, CG)

‘So, we do have those guidelines; even though the TB guidelines are there, they have some information on how to screen, how to manage. We just have to refer and check under the management of TB during pregnancy.’ (P6, F, CC)

#### Counselling and health education

Counselling and health education enable pregnant women to make informed choices regarding their health concerns. The participants reported that they offered women counselling and health education before conducting TB screenings on them. These quotations lend credence to this:

‘[*A*]fter that, do we screen them before anything else? We provide them with general counselling. We provide them with TB counselling and health education.’ (P3, F, CF)

‘We do counselling, health education, we educate the patient about TB infection as a whole and explain why we are focusing on screening for TB.’ (P1, F, CE)

### Tuberculosis screening challenges

Participants raised a range of difficulties they frequently face while doing their tasks, which include screening for TB and managing HIV-positive pregnant women in their facilities. Three themes emerged: shortage of working tools, withholding of TB information, and language barrier.

#### Shortage of working tools

A questionnaire with TB screening questions serves as the screening tool. Participants expressed their displeasure at how they occasionally run out of the tool they use when screening for TB in pregnant women. The reply that follows demonstrates:

‘The shortage of equipment … at times you are required to use [*a*] TB screening tool, and there aren’t any screening tools.’ (P9, F, CB)

‘I think if we can have the screening tools, making copies, and each and every pregnant woman, we give it to them, so that even if, when they are at home, they will be able to screen themselves.’ (P5, F, CD)

#### Withholding of tuberculosis symptoms information

Participants expressed worries that some pregnant women choose not to provide the necessary information concerning TB symptoms during history collection, which may cause them problems. Here is a quote from the participant to vouch for it:

‘I’m not sure if the patient is being obstinate or if she believes we will take her in some other way, but she simply … she just decided to be silent. Alternatively, she might believe she has a flu that will eventually go off.’ (P6, F, CC)

‘Concerning TB screening, I can say that the patient may have those symptoms … and that the client may conceal certain information.’ (P8, F, CF)

#### Language barrier to international women

One of the problems regarding TB screening among pregnant women was also listed as communication issues. Participants acknowledged the fact that occasionally foreign pregnant women find it difficult to converse in South African languages. The participants said the following:

‘Is where we can leave the patient because if they cannot provide us with a relevant response or if they don’t grasp what we are asking, then that is where we will be losing the patient.’ (P6, F, CC)

‘Another thing might be language break down, we are having those people that are coming from Zimbabwe, who might come to the clinic being alone, she does not understand English, she does not understand Sepedi.’ (P4, F, CA)

## Discussion

The study aimed to explore midwives’ challenges regarding TB screening in pregnant women. The study revealed that midwives understand their roles and responsibilities regarding TB screening among pregnant women.

The study’s findings indicated that TB screening is performed on each expectant patient who visits the clinic. This aligns with the study by Miele, Bamrah Moris and Tepper (2020:4), emphasising the importance of thoroughly evaluating and screening all pregnant women who come for antenatal care booking for TB through assessing symptoms, physical examinations, and asserting TB risk factors. However, routine screening of pregnant women is still underutilised, as observed by Vijayageetha et al. (2018:7). Based on the study’s findings, it is assumed that pregnant women who visit for their antenatal appointments are provided TB screening.

This study revealed confirmation supporting the application of and compliance with guidelines for detecting and managing TB in pregnant women. Most of the participants stated that they refer to TB guidelines, prevention of mother to child transmission (PMTCT) guidelines, basic antenatal care (BANC) guidelines, and essential drug lists (EDL)s when needed. The results support the claim made by Ebben et al. (2017:5) that materials and educational possibilities paired with assessment and feedback improve adherence to guidelines. It is noteworthy from the results that midwives at PHC clinics have access to and follow a variety of guidelines that direct TB screening in pregnant women.

To help pregnant women understand the value of TB screening, participants reported that counselling and health education were provided during the TB screening (Mutabazi et al., 2020:14). Hahn and Truman (2015:12) confirmed the benefits of counseling and health education. According to Hahn and Truman’s study (2015:12) and Kabir (2017:22), health education is a key social factor affecting a community’s health. The claim is that the midwives recognise the value of counselling and health education for pregnant women so that they may give informed consent for TB screening.

Participants in this study identified a challenge to providing high-quality TB screening services to pregnant women as the lack of screening tools renders TB screening less effective among pregnant women. Similar conclusions were drawn from the study by Christian, Smith and Hompashe (2018:729) and Hildingsson, Westlund and Wiklund (2013:88), which showed that TB screening among pregnant women in South Africa is not as effective as it should be considering the lack of resources such as screening tools at most institutions. Kotze (2018:159) asserted that workplace resources significantly improve excitement and dedication: two factors in work engagement. For midwives to provide proper TB screening and prevent TB among pregnant women, they must be given adequate screening tools.

The study further revealed that midwives face challenges and difficulties when providing TB screening services to pregnant women. The study’s findings indicate that pregnant women occasionally decide not to provide midwives with important information on TB screening, which may delay an early diagnosis. Similar findings of pregnant women not providing midwives with information have been reported in recent studies (Elwell 2016:973). According to Bashorun et al. (2020:10), pregnant women in rural areas are believed to spread misleading information about TB owing partly to negative perceptions Thus, there is a need to enhance health education about TB screening during pregnancy to improve the public’s knowledge of the disease’s risk in pregnant women.

The findings highlighted that participants occasionally encounter difficulties when pregnant women arrive for antenatal care, and they are unable to express themselves in SA languages. The participant claimed that because the woman could not comprehend the spoken language, they ultimately decided to omit the TB screening part. According to research by Al Shamisi et al. (2020:27) and Azam and Watson (2017:1158), language barrier significantly contributes to miscommunication between midwives and patients. As it is clear from this study that midwives cannot screen TB in pregnant women who do not understand South African languages, it is necessary to teach them several fundamental languages.

The results imply that participants knew that every antenatal care visit should include a TB screening for every pregnant woman. However, they do not provide such services because of several reasons and challenges shared. According to the PMTCT Guideline, all pregnant women should be screened for TB at each antenatal care visit (NDoH 2023:26). This concurs with the recommendation by the WHO (2017:4). Therefore, omitting TB screening among pregnant women could lead to delayed detection and severe complications such as maternal and neonatal mortality from untreated TB.

### Strengths and limitations

The study was conducted in a locality where maternal death from diseases unrelated to pregnancy was most prevalent, with TB being the main cause. The Limpopo province in South Africa was the sole location of the study. As a result, the results cannot be extrapolated to other provinces.

### Recommendations

Based on the study’s findings, the researcher recommends the following:

- The provision of functional equipment, in this case, the TB screening tools, to screen for TB consistently and successfully in pregnant women should be prioritised.
- For better communication during TB screening, midwives should be taught the fundamental languages of other nations, especially Zimbabwean and Mozambican.
- The facilities must foster a culture of health education regarding the risk of TB infection during pregnancy through tribal authorities, community events, and radio spots to enhance knowledge and understanding of TB screening during pregnancy. Pregnant women will know the services they should receive during each visit.
- The study might be expanded to other localities, districts, and SA provinces to better understand the need for TB screening during pregnancy.

## Conclusion

The study’s findings suggest that midwives know their obligations while performing antenatal TB screenings. However, they are unable to provide the best TB screening services to pregnant women because of several obstacles. As a result, it is advised that the Department of Health provides midwives the tools and training they need to perform at their best. As the study was limited to a single locality in the province of Limpopo, it can be expanded to include other provinces in South Africa to gain a deeper knowledge of the occurrence.
